# Addressing System and Clinician Barriers to Emergency Department-initiated Buprenorphine: An Evaluation of Post-intervention Physician Outcomes

**DOI:** 10.5811/westjem.18320

**Published:** 2024-04-09

**Authors:** Jacqueline J. Mahal, Polly Bijur, Audrey Sloma, Joanna Starrels, Tiffany Lu

**Affiliations:** * Jacobi Medical Center, Department of Emergency Medicine, Bronx, New York; † Albert Einstein College of Medicine, Department of Emergency Medicine, Bronx, New York; ‡ Albert Einstein College of Medicine/Montefiore Medical Center, Department of Medicine, Bronx, New York; § Jacobi Medical Center, Department of Psychiatry & Behavioral Sciences, Bronx, New York

## Abstract

**Introduction:**

Emergency departments (ED) are in the unique position to initiate buprenorphine, an evidence-based treatment for opioid use disorder (OUD). However, barriers at the system and clinician level limit its use. We describe a series of interventions that address these barriers to ED-initiated buprenorphine in one urban ED. We compare post-intervention physician outcomes between the study site and two affiliated sites without the interventions.

**Methods:**

This was a cross-sectional study conducted at three affiliated urban EDs where the intervention site implemented OUD-related electronic note templates, clinical protocols, a peer navigation program, education, and reminders. Post-intervention, we administered an anonymous, online survey to physicians at all three sites. Survey domains included demographics, buprenorphine experience and knowledge, comfort with addressing OUD, and attitudes toward OUD treatment. Physician outcomes were compared between the intervention site and the control sites with bivariate tests. We used logistic regression controlling for significant demographic differences to compare physicians’ buprenorphine experience.

**Results:**

Of 113 (51%) eligible physicians, 58 completed the survey: 27 from the intervention site, and 31 from the control sites. Physicians at the intervention site were more likely to spend <75% of their work week in clinical practice and to be in medical practice for <7 years. Buprenorphine knowledge (including status of buprenorphine prescribing waiver), comfort with addressing OUD, and attitudes toward OUD treatment did not differ significantly between the sites. Physicians were 4.5 times more likely to have administered buprenorphine at the intervention site (odds ratio [OR] 4.5, 95% confidence interval 1.4–14.4, *P* = 0.01), which remained significant after adjusting for clinical time and years in practice, (OR 3.5 and 4.6, respectively).

**Conclusion:**

Physicians exposed to interventions addressing system- and clinician-level implementation barriers were at least three times as likely to have administered buprenorphine in the ED. Physicians’ buprenorphine knowledge, comfort with addressing and attitudes toward OUD treatment did not differ significantly between sites. Our findings suggest that ED-initiated buprenorphine can be facilitated by addressing implementation barriers, while physician knowledge, comfort, and attitudes may be harder to improve.

Population Health Research Capsule What do we already know about this issue?

*While ED-initiated buprenorphine for the treatment of opioid use disorder has increased, system- and clinician-level barriers continue to limit its use.*

What was the research question?

*We sought to compare whether interventions addressing barriers to ED-initiated buprenorphine would improve administration of buprenorphine.*

What was the major finding of the study?

*Physicians at the intervention site were 4.5 times more likely to have administered buprenorphine (95% CI 1.4–14.4,* *P = 0.01).*

How does this improve population health?

*ED-initiated buprenorphine can be facilitated by addressing both system- and clinician-level barriers, although physician knowledge, comfort, and attitudes may be harder to improve.*

## INTRODUCTION

Opioid-related overdose deaths in the US have increased since the 1990s, and in the 12 months ending June 2023 provisional overdose deaths exceeded 81,000.^1^ The emergency department (ED) has been involved in addressing the opioid crisis by implementing opioid-sparing pain management protocols and treating opioid overdoses. Yet patients with non-fatal unintentional opioid overdose visits to the ED are still 100 times more likely to die of an overdose within a year of their index visit than those from a demographically matched population.^2^ Emergency departments are in the unique position to initiate and link to evidence-based treatment for opioid use disorder (OUD) when a patient presents acutely with opioid withdrawal or non-fatal overdose.

Buprenorphine, a partial opioid agonist, is an effective medication to treat OUD that has historically not been offered in ED settings. In 2015, D’Onofrio et al published a seminal, randomized controlled study demonstrating the efficacy of ED-initiated buprenorphine and ongoing engagement in OUD treatment at 30-days post discharge.^3^ Follow-up studies also demonstrated that ED-initiated buprenorphine is an effective intervention, with ongoing OUD treatment at 30 days in 50–86% of the patients.^4^^,^^5^ On the heels of these findings, the Substance Abuse and Mental Health Services Administration published a resource guide in 2021 acknowledging the ED as an important site for provision of OUD treatment.^6^ In the same year, the American College of Emergency Physicians published consensus recommendations for OUD treatment including use of buprenorphine in the ED.^7^

While buprenorphine use in the ED has increased in recent years,^8^ multiple barriers at the system and clinician level limit the implementation of ED-initiated buprenorphine.^9^^–^^13^ System-level barriers include lack of streamlined order sets for OUD treatment, difficulty referring to ongoing treatment services after discharge, limited availability of expert physicians and pharmacists for consultation, and lack of access to dedicated care coordinators, social workers, or peer counselors. Clinician-level barriers include lack of knowledge, comfort and experience with buprenorphine and OUD treatment, a historical need for a buprenorphine prescribing waiver,^14^ as well as stigma toward patients with OUD.

Few studies have examined specific interventions that address clinician-level barriers and post-intervention clinician outcomes. Foster et al described a financial incentive program for emergency physicians to complete the then-required buprenorphine waiver training and reported a positive but variable increase in buprenorphine prescribing in the five months after the incentive.^14^ Butler et al reported on a set of behavioral-science informed interventions that increased physician initiation of OUD-related treatments^15^ at a single academic ED site with a robust addiction clinic program. Khatri et al randomized physicians to a clinician-level intervention of either a didactic-only group or a didactic plus weekly messaging and a financial incentive group.^16^ While 33% of all participants prescribed buprenorphine for the first time in the 90 days post-intervention, buprenorphine administration frequency or knowledge did not differ significantly between the groups. In an ongoing, multicenter effectiveness study of buprenorphine initiation in the ED, D’Onofrio et al described multiple system-level implementation facilitators that include clinical protocols, learning collaboratives, and referral programs.^17^^,^^18^ The implementation facilitation period was associated with a higher number of emergency clinicians who completed the buprenorphine prescribing waiver, as well as ED visits where clinicians prescribed buprenorphine and naloxone.^19^

We contribute to the growing body of literature by describing a set of interventions that addressed multiple system- and clinician-level implementation barriers to ED-initiated buprenorphine in a safety-net ED. We evaluated post-intervention physician outcomes and compared these between the ED site with targeted interventions and two related sites without targeted interventions. Our aim was to determine whether addressing multiple implementation barriers to ED-initiated buprenorphine is associated with improved buprenorphine knowledge, comfort with addressing OUD, and attitudes toward OUD treatment among physicians at the intervention site. We hypothesize that physicians at the intervention site had improved experience with administering buprenorphine in the ED.

## METHODS

### Study Design

We conducted a cross-sectional study of attending physicians at three EDs affiliated with a large, urban emergency medicine (EM) residency program. Physician knowledge, comfort with, and attitudes toward OUD treatment, as well as experience with administering buprenorphine in the ED, were compared between one intervention site (where a multifaceted set of interventions aimed at addressing clinician- and system-level barriers to ED-initiated buprenorphine was implemented) vs two control sites (where interventions focused on ED-initiated buprenorphine were not implemented). The study was approved by the affiliated institutional review boards (IRB#2019-10920).

### Setting

This study took place at three EDs affiliated with a large academic EM training program with 84 residents per year and 100 full-time attending physicians on faculty. One ED site is part of the New York City municipal hospital system, while the other two ED sites are part of a large, private, academic health system. All three EDs see a high visit volume around 70,000 per annum per site and provide safety-net care to a payor mix that is predominantly publicly insured. All three EDs are in the borough of The Bronx, New York, where the opioid-related overdose rate was 73.6 per 100,000 in 2022, representing the highest of all five boroughs in New York City.^20^ Consistent with most EM practices across the country, the three ED training sites have not historically offered buprenorphine for opioid withdrawal and OUD treatment.

#### Intervention Site

Between November 2018–June 2020, the municipal hospital-based ED site (herein referred to as “intervention site”) implemented a multifaceted set of interventions to address system-and clinician-level barriers to ED-initiated buprenorphine. System-level interventions customized for the ED included the following: 1) an electronic health record (EHR) note template for opioid withdrawal and OUD assessment; 2) a clinical protocol for administering buprenorphine in the ED; 3) a clinical workflow to provide naloxone training and take-home kits for overdose prevention; and 4) a peer navigation program to facilitate referral and linkage to outpatient buprenorphine treatment, including an in-house substance use disorder treatment program. System-level interventions were funded and developed by a centralized leadership team from the municipal public hospital system. Local ED implementation was facilitated by a clinician champion (JM) who worked closely with an interdisciplinary team of emergency medicine, behavioral health, pharmacy, and social work leadership. Initial salary support for this work was grant-funded.

Clinician-level interventions included the following: 1) a modest financial incentive for voluntary completion of buprenorphine waiver training and obtaining the prescribing waiver; 2) regular updates and reminders about system-level interventions at EM faculty meetings every two weeks; and 3) two, one-hour grand rounds lectures that reviewed the evidence for ED-initiated buprenorphine and the availability of clinical protocols to support buprenorphine treatment. Grand rounds lectures at the time of intervention were conducted in person and voluntarily attended by faculty and residents across the EM residency program. Many of the interventions were introduced in an overlapping manner and refined iteratively during the two-year implementation period.

#### Control Site

During the same period, a clinical protocol and an order set to support hospital-initiated buprenorphine were also being implemented at the two other ED sites based at the private, academic health system (referred to as “the control site”); however, these interventions did not focus on the ED. Peer navigators based in the ED were available but were not dedicated to support referral and linkage to outpatient buprenorphine treatment. Neither were financial incentives for completion of buprenorphine waiver training or physician meetings dedicated to ED-initiated buprenorphine offered.

### Participants

We recruited study participants based on the following criteria: 1) licensed physician eligible to obtain a waiver to prescribe buprenorphine; and 2) attending physicians practicing at either the intervention or control site. We did not include resident physicians in our sample because they rotate at both the intervention and control sites and would have experienced variable exposure to the interventions aimed at ED-initiated buprenorphine. Neither did we include physician assistants who are an important part of the EM workforce because they did not receive the financial incentive and did not attend faculty meetings or grand rounds where most of the clinician-facing interventions occurred.

### Data Collection

Between September–December 2020, we emailed 113 eligible emergency physicians at the three ED sites to introduce the opt-in study and continued to send monthly email reminders. We also announced the study in person at attending physician meetings at two of the three sites that could allocate meeting time during the COVID-19 public health emergency. Individualized email reminders were sent to attending physicians at all sites in the last month of study recruitment. The survey was administered anonymously in English using the online platform Qualtrics (Qualtrics, Provo, UT). Upon completion of the questionnaire, participants were eligible to enter a raffle to win one of five $50 gift cards.

A 22-item survey was adapted from previously published research on clinician barriers to buprenorphine prescribing.^9^^,^^11^ The survey instrument we developed consisted of five domains: demographics; buprenorphine experience; buprenorphine knowledge; comfort with addressing OUD; and attitudes toward OUD treatment.

Self-reported demographics included age, gender, race, ethnicity, years in practice, and amount of time spent working clinically (clinical time). The number of years in practice was measured by the number of years since American Board of Emergency Medicine certification date, and respondents were considered junior attending physicians if they had seven or fewer years in practice. Clinical time was a dichotomous measure of less than vs ≥ 75%, based on the rationale that attending physicians who spend <75% clinical time represent clinician-educators, researchers, or administrators.

For buprenorphine experience, participants were asked to answer yes/no to ever administering buprenorphine in the ED, completing the buprenorphine waiver training, and receiving their buprenorphine prescribing waiver. Buprenorphine knowledge was evaluated with seven questions specific to the clinical use of buprenorphine using a three-point Likert scale (“agree-neutral-disagree”), where agreeing or disagreeing correctly to the knowledge questions was a key outcome. Comfort with OUD treatment was also evaluated with a three-point Likert scale (“comfortable-somewhat comfortable-not comfortable”) regarding management of opioid withdrawal, response to opioid overdose, counseling on and administering medications for OUD, and referral to outpatient treatment for substance use disorder. Attitudes toward OUD treatment were measured with level of agreement (“agree-neutral-disagree”) to stigmatizing statements describing patients with OUD as difficult to treat, buprenorphine as substituting one drug for another, and prescribing buprenorphine for OUD as increasing medicolegal risk.

### Data Analysis

We calculated descriptive statistics for demographic characteristics, buprenorphine experience, and buprenorphine knowledge for physicians at the intervention and control sites. Fisher exact tests were used to assess whether physicians’ demographic characteristics and buprenorphine experience differed by site. We examined buprenorphine knowledge by calculating a composite knowledge score based on the number of correct answers to the seven knowledge questions and compared them by site using the Mann-Whitney U-test. Physicians’ comfort with addressing OUD and attitudes toward OUD treatment are described with proportion of responses with “comfortable” and “agree,” and compared by site with Fisher exact tests and Fisher-Freeman-Halden tests, for variables with more than two categories.

We conducted a post-hoc multivariable analysis because of a statistically significant difference between physicians’ buprenorphine experience of “ever administered buprenorphine” by site. We examined possible confounding of this association by the demographic characteristics that are significantly associated with the site. We used logistic regression to assess the association between buprenorphine administration and site while controlling for these covariates. Following the recommendation that one variable should be used for every 10 participants with the outcome, we ascertained that only two variables could be included in a single analysis as there were 20 participants who had “ever administered buprenorphine.” Thus, we ran analyses with site and each of the possible confounders separately. All tests were two-sided with a statistical significance criterion of 0.05. We used SPSS version 27 (IBM Corp, Armonk, NY) for all statistical analyses.

## RESULTS

Among the 113 eligible attending physicians, 58 (51.3%) physicians fully completed the survey, with 27 responses from the intervention site and 31 responses from the control site. As shown in Table 1, no significant differences in the demographic characteristics of gender, race, and ethnicity were found among emergency physicians by site. Physicians were more likely to spend <75% of time in clinical practice at the intervention vs control sites, 44.4% vs 19.4%, respectively (*P* = 0.05). Nearly twice as many physicians at the intervention site were in clinical practice for seven years or less compared to those at the control site, 70.4% vs 38.7%, respectively (*P* = 0.02). In other words, physicians at the intervention site were more likely to be clinician-educators, researchers and administrators, and junior attending physicians.

**Table 1.** Demographic, experience, and knowledge participant characteristics.

	Intervention site N = 27 n (%)	Control sites N = 31 n (%)	*P*-value
Demographic characteristics			
Gender			0.50
Female	14 (51.9)	13 (41.9%)	
Male	11 (40.7)	17 (54.8%)	
Decline to answer	2 (7.4%)	1 (3.2%)	
Race			0.69
White	17 (63.0%)	22 (75.9%)	
Black	3 (11.1%)	3 (10.3%)	
Asian	2 (3.7%)	3 (10.3%)	
Other	2 (7.4%)	1 (3.4%)	
Decline to answer	4 (14.8%)	1 (3.4%)	
Ethnicity			0.68
Hispanic or Latina/o	2 (7.4%)	4 (12.9%)	
Not Hispanic or Latina/o	25 (92.6%)	27 (87.1%)	
Clinical time			0.05
<75%	12 (44.4%)	6 (19.4%)	
75%+	15 (55.6%)	25 (80.6%)	
Years in practice			0.02*
>7 years	8 (29.6%)	19 (61.3%)	
≤7 years	19 (70.4%)	12 (38.7%)	
Buprenorphine experience			
Ever administered buprenorphine			0.01*
Yes	14 (51.9%)	6 (19.4%)	
No	13 (48.1%)	25 (80.6%)	
Completed waiver training			0.80
Yes	15 (55.6%)	16 (51.6%)	
No	12 (44.4%)	15 (48.4%)	
Obtained prescribing waiver among those who completed waiver training			0.74
Yes	12 (80.0)	12 (75.0)	
No	3 (20.0)	4 (25.0)	
Buprenorphine knowledge			
Mean (SD) number of correct responses (7 items)	3.4 (2.0)	3.3 (2.1)	0.82
Median (range)	3 (0–7)	4 (0 6)	0.88

^*^ Statistical significance with p-value for comparison (p < .05).

**Figure 1.**
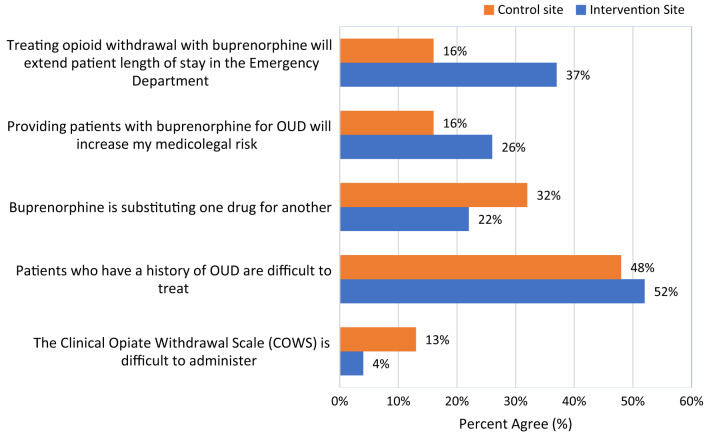
Physician attitudes towards patients living with opioid use disorder (OUD)^1^ and use of buprenorphine by site (percent agree.) Physician agreement with the statements along the vertical axis by site. No statistical difference found.

**Figure 2.**
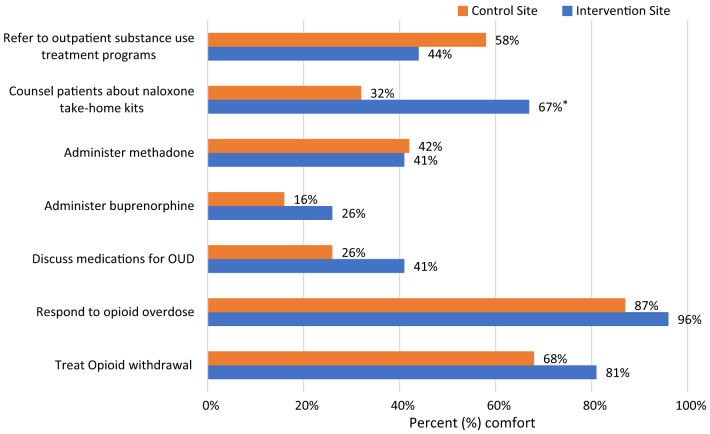
Physician percent comfort with addressing opioid use disorder by site. Physician comfort with the activities listed along the vertical axis by site. *Statistical significance with *P*-value for comparison (*P* < .05).

For buprenorphine experience, more physicians at the intervention site reported “ever administered buprenorphine” in their clinical practice than physicians at the control site, 51.9% vs 19.4%, respectively (*P* = 0.01). Over half of the physician respondents completed the waiver training at both the intervention and control sites, 55.6% and 51.6%, respectively. Of those who completed the waiver training, most physicians obtained the prescribing waiver. There was no statistical difference in waiver training completion and status by site. For buprenorphine knowledge, the median score of correct answers (of the seven knowledge questions) was three for physicians at the intervention site, which was similar to the median score of four at the control site (Mann-Whitney U = 428, *P* = 0.88). As seen in Figures 1 and 2, physicians’ comfort with addressing OUD and their attitudes toward OUD treatment did not differ significantly between the intervention and control sites.

The post-hoc analysis (see Table 2) of the association between buprenorphine administration and site indicates that physicians at the intervention site were 4.5 times more likely to have administered buprenorphine than those at the control site (OR 4.5, 95% CI 1.4 – 14.4, *P* = 0.01). After adjusting for the two demographic characteristics that differed by site (clinical time and years in practice), the likelihood of buprenorphine administration remained high and statistically significant among physicians at the intervention site compared to the control site (OR 3.5 with clinical time controlled, 4.6 with years in practice controlled, respectively).

**Table 2.** Predictors of buprenorphine administration by physician characteristics.

	Model 1 Univariate	Model 2 Site and clinical time	Model 3 Site and years in practice
	OR (95% CI)	OR (95% CI)	OR (95% CI)
Site Intervention Control	4.5 (1.4–14.4)^*^ Ref	3.5 (1.0–12.0)^*^ Ref	4.6 (1.3–15.8)^*^ Ref
Clinical time <75% 75%+	5.4 (1.6–18.0)^*^ Ref	4.3 (1.2–15.0)^*^ Ref	
Years in practice ≤7 years >7 years	1.5 (0.5–4.5) Ref		0.9 (0.3–3.2) Ref

^*^ Statistically significant *P*-value < 0.05.

*Ref*, reference group; *OR*, odds ratio.

## DISCUSSION

In this study, we found that emergency physicians who were exposed to a multifaceted set of interventions that addressed system- and clinician-level barriers to ED-initiated buprenorphine at their clinical site were at least three times as likely to have administered buprenorphine after adjusting for clinical time and years in practice. Yet physicians’ buprenorphine knowledge, comfort with addressing OUD, and attitudes toward OUD treatment did not differ significantly between the intervention and control sites. Our findings suggest that ED-initiated buprenorphine can be facilitated by addressing system-level implementation barriers, while clinician knowledge, comfort, and attitudes may be harder to improve and may require long-term and/or different interventions.

The system-level interventions described were a series of tools and services introduced to the ED by interdisciplinary stakeholders to encourage the use of evidence-based, ED-initiated buprenorphine that had not previously been considered standard treatment for patients living with OUD. Integrated EHR templates and clinical protocols and workflows were tools to support clinical decision-making, while the peer navigation program provided harm reduction interventions and supported post-discharge planning and linkage to care. The implementation of these system-level interventions was intended to minimize the burden on clinicians and to reduce variation in care.^21^ The impact of each intervention was not measured individually because many components were introduced and refined in an overlapping, iterative manner during the implementation period. (For example, announcements and education regarding the EHR order sets and clinical protocols occurred at a similar time and across subsequent meetings.) The cross-sectional study captured only clinician outcomes after receiving the whole set of system-level interventions, which is a limitation of measuring real-world implementation facilitation.

Implementation of these system-level tools and services required interventions at the clinician level to introduce, familiarize, and remind clinicians of available tools and support services. Frequent reminders, educational opportunities, financial incentives for the then-required buprenorphine prescribing waiver coursework were an attempt to encourage knowledge of and comfort with ED-initiated buprenorphine with the goal to support a change in clinical practice to *treat* OUD, not just respond to acute overdoses, in the ED. Our clinician-level interventions eased the implementation of system-level interventions in a similar manner, as the behavioral science-based “nudges” were used to increase the number of physicians who obtained a waiver at another urban, academic ED.^22^ The same group also used clinician-level nudges in the form of best practice advisories in the EHR and monthly emails to increase the use of ambulatory referrals to a Bridge Clinic and buprenorphine administration.^15^ An important part of the process appears to include a clinical champion who can work with stakeholders to overcome institutional barriers^18^^,^^19^^,^^23^ to refine workflows and protocols, and who can also be a content expert resource to colleagues to introduce evidence-based practice updates and reminders.

In our study, physicians’ clinical time and years in practice had an impact on the likelihood of practicing ED-initiated buprenorphine. Clinical time in practice is a variable used to differentiate between physicians with or without dedicated time for clinical education, research, and administration, which was hypothesized to have an independent effect on adoption of emerging clinical practices. Years in independent clinical practice is used as a measure to account for secular trends in EM training; attending physicians with fewer than seven years in clinical practice may have been exposed to frequent press on the opioid epidemic and changing guidelines for OUD treatment in the ED. Im et al report that junior emergency physicians are more likely to view OUD as a chronic disease and approve of buprenorphine initiation in the ED,^24^ even if junior emergency physicians expressed a similar sense of frustration treating patients with OUD as senior physicians. Our study did not include resident physicians to minimize cross-contamination of exposure to interventions. Other studies have found that emergency physicians in their residency training are eager to implement ED-initiated buprenorphine.^15^^,^^22^ Attitudes among emergency physicians are generally changing toward OUD, and it is increasingly being viewed as a chronic disease with acute manifestations that should be treated in the ED setting.^24^^,^^25^

The removal of the buprenorphine-prescribing waiver requirement is an acknowledgment that this clinician-level barrier impeded access to treatment for OUD.^26^^,^^27^ While this study was completed at a time when the buprenorphine-prescribing waiver requirement was still in effect (and justified financial incentives for emergency clinicians who voluntarily obtained a prescribing waiver), we expect that future interventions to address clinician-level barriers to buprenorphine initiation in the ED will still require a clinical champion who can regularly provide updates about implementation and lead education efforts.

## LIMITATIONS

Limitations to our study include a relatively small sample size with a 58% response rate, which may have contributed to a sampling bias. Our clinical sites are in an urban area with a high prevalence of opioid overdose and OUD, which may influence physician interest in and knowledge of OUD and, thus, participation in the survey. Implementation of ED-initiated buprenorphine at the intervention site received financial support and departmental resources in a tertiary-care municipal hospital as well as initial grant funding for salary support of the clinical champion, which may limit generalizability to ED settings in smaller, rural and/or under-resourced hospitals. Without pre-/post-evaluations for each intervention, we were unable to assess whether a particular intervention influenced the difference in buprenorphine administration at the intervention site. Buprenorphine experience is self-reported; responses regarding buprenorphine administration in the ED are not linked to pharmacy data from the interventional or control sites. Lastly, cross-contamination of attending physicians’ exposures to interventions may have occurred via residents who rotate among the intervention and control sites. It may have also occurred at the grand rounds lectures where all faculty from the residency sites are invited; however, total faculty attendance typically hovered below 10% for the then in-person lectures.

## CONCLUSION

Our study compares the administration of ED-initiated buprenorphine at two similar and related ED settings where physicians at one site were exposed to a multifaceted set of interventions to ED-initiated buprenorphine. Physicians exposed to interventions designed to address system- and clinician-level barriers were more likely to initiate buprenorphine for OUD treatment in their clinical practice. Future implementation efforts should examine interventions that are tailored to implementation barriers even after the buprenorphine- prescribing waiver requirement has been eliminated, including residency education to improve the understanding and uptake of ED-initiated buprenorphine. Coupling pharmacy-level buprenorphine administration and prescribing data with physician-reported outcomes will also help parse out impact of future interventions.
